# CONNECTING OLDER ADULTS AND DOCTOR OF PHYSICAL THERAPY STUDENTS THROUGH A COMMUNITY-BASED EXERCISE PROGRAM

**DOI:** 10.1093/geroni/igad104.3061

**Published:** 2023-12-21

**Authors:** Gabriele Moriello, Alice Hamilton, Julie Bush, Michelle Nunno-Evans, Shannon Schoellig, Nancy Hollins

**Affiliations:** Utica University, Utica, New York, United States; Utica University, Utica, New York, United States; Utica University, Utica, New York, United States; Utica University, Utica, New York, United States; Utica University, Utica, New York, United States; Utica University, Utica, New York, United States

## Abstract

The purpose of this qualitative study was to explore the perspectives of older adults regarding their experience participating in an intergenerational Integrated Clinical Experience (ICE) with Doctor of Physical Therapy (DPT) students. This ICE was designed as a community-based exercise program for older adults. DPT students designed individualized one-hour fitness plans for participants every week over the course of a semester. To obtain the perspectives of older adults, a descriptive phenomenological approach was chosen. A purposive sample of 16 older adults involved in the wellness program participated in 6 focus groups and one interview. Results of the thematic analysis revealed six themes; Blending of the Generations, It Motivated Me, I Can Do It Now, Mental and Social Boost, Program Tailored to Me, and Learn and Modify Habits. Participant interviews highlighted how older adults highly valued the relationships with students, and how the intergenerational environment of the program benefited their physical, social, and mental well-being. They also noted how students served as a source of motivation, leading to behavioral change and improvements in functional outcomes. These findings support existing literature on intergenerational programs, but this is one of the few studies that looked specifically at an intergenerational ICE. This ICE provided an innovative manner to meet the physical challenges faced by many older adults, and the intergenerational nature of the experience may have contributed to the well-being of all those involved. Future research using quantitative methods are needed to extend what was learned beyond the specific context of this study.

